# Can COVID-19 impact the natural history of paracoccidioidomycosis? Insights from an atypical chronic form of the mycosis

**DOI:** 10.1590/S1678-9946202466057err

**Published:** 2024-01-05

Rev Inst Med Trop Sao Paulo. 2023;65:e57

http://doi.org/10.1590/S1678-9946202365057

On page 1:

Where it reads: Nathan Costa Marques

Should be read: André Nathan Costa 0000-0002-8025-6940

